# Systematic surveillance for management of Dioxin contamination risks in the food chain: Germany and related cases

**DOI:** 10.1186/1751-0147-54-S1-S3

**Published:** 2012-02-24

**Authors:** Abraham Brouwer, Peter Benisch

**Affiliations:** 1 BioDetection Systems BV, Science Park 406, 1098 XH Amsterdam, The Netherlands; 2 Institute of Animal Ecology, VU University Amsterdam, The Netherlands

## 

Environmental pollutants, such as polychlorinated dibenzo-p-dioxins (PCDDs), furans (PCDFs) and dioxin like PCBs keep appearing in the food chains due to their recalcitrant environmental characteristics and due to practices of raw materials, feed and food producers. Despite emission control measures taken, which have resulted in significant reductions in environmental contamination, Dioxin-food crises have appeared and still appear in the media almost every year, or sometimes several times a year since the beginning of the 21^st^ century. In the late years of the last century, the Belgian chicken crisis, lasted for more than half a year regarding import/export bans among EU countries and with non-European countries. The total economic costs of this crisis exceeded billion Euro. Since then the European Commission has established a set of instruments, including a rapid alert system, tight control measures and maximum limit values in food , feed and raw materials within the EU and for imported goods and products imported from non-EU countries. The development, introduction, and EU acceptance of fast and high capacity screening bioassays, such as CALUX provide for a methodology that can quickly assess the extent of the contamination, and therefore can limit the spreading of the problem and allow the early market release of food, feed and related products once they have been cleared by the fast screening tool. Nevertheless, the introduction of fast bio-analytical tools by various EU member countries and in the World is slow, mostly due to a variety of barriers (acceptance, psychological, competition) well-known to exist for introduction of novel technologies. However, in food crisis situations, affected countries show a high willingness to quick adoption and implementation of fast screening methods, in order to provide for sufficient analytical capacity and to limit the economic damages due to e.g., prolonged export bans. In this presentation the use and application of CALUX as a fast screening method is given, as well as its application in several food crisis cases will be presented, including the recent German Dioxin crisis in 2011.

